# Genomic Scan of Male Fertility Restoration Genes in a ‘Gülzow’ Type Hybrid Breeding System of Rye (*Secale cereale* L.)

**DOI:** 10.3390/ijms22179277

**Published:** 2021-08-27

**Authors:** Nikolaj Meisner Vendelbo, Khalid Mahmood, Pernille Sarup, Peter Skov Kristensen, Jihad Orabi, Ahmed Jahoor

**Affiliations:** 1 Nordic Seed A/S, Grindsnabevej 25, 8300 Odder, Denmark; khma@nordicseed.com (K.M.); pesa@nordicseed.com (P.S.); pskr@nordicseed.com (P.S.K.); jior@nordicseed.com (J.O.); ahja@nordicseed.com (A.J.); 2 Department of Agroecology, Faculty of Technology, Aarhus University, Forsøgsvej 1, Flakkebjerg, 4200 Slagelse, Denmark; 3 Department of Plant Breeding, The Swedish University of Agricultural Sciences, 23053 Alnarp, Sweden

**Keywords:** restoration of male fertility (*Rf*), cytoplasmic male sterility (CMS), pentatricopeptide repeat protein (PPR), mitochondrial transcription termination factor (mTERF), 600K SNP array, genome-wide association study (GWAS), RNAseq, chi-square, linkage disequilibrium

## Abstract

Efficient and stable restoration of male fertility (Rf) is a prerequisite for large-scale hybrid seed production but remains an inherent issue in the predominant fertility control system of rye (*Secale cereale* L.). The ‘Gülzow’ (G)-type cytoplasmic male sterility (CMS) system in hybrid rye breeding exhibits a superior Rf. While having received little scientific attention, one major G-type *Rf* gene has been identified on 4RL (*Rfg1*) and two minor genes on 3R (*Rfg2*) and 6R (*Rfg3*) chromosomes. Here, we report a comprehensive investigation of the genetics underlying restoration of male fertility in a large G-type CMS breeding system using recent advents in rye genomic resources. This includes: (I) genome-wide association studies (GWAS) on G-type germplasm; (II) GWAS on a biparental mapping population; and (III) an RNA sequence study to investigate the expression of genes residing in Rf-associated regions in G-type rye hybrids. Our findings provide compelling evidence of a novel major G-type non-PPR *Rf* gene on the 3RL chromosome belonging to the mitochondrial transcription termination factor gene family. We provisionally denote the identified novel *Rf* gene on 3RL *RfNOS1.* The discovery made in this study is distinct from known P- and C-type systems in rye as well as recognized CMS systems in barley *(Hordeum vulgare* L.) and wheat (*Triticum aestivum* L.). We believe this study constitutes a stepping stone towards understanding the restoration of male fertility in the G-type CMS system and potential resources for addressing the inherent issues of the P-type system.

## 1. Introduction

In recent years, hybrids have become the predominant class of cultivated winter rye (*Secale cereale* L.) in Northern Europe [1]. Outperforming population-based cultivars, hybrids in rye demonstrate strong heterotic effects on all developmental and yield characteristics [2,3]. Breeding of hybrids relies on the existence of cytoplasmic male sterility (CMS) and restoration of male fertility (Rf) genes that reside in genetically distinct parental populations [3,4]. This system efficiently enables control of parental crossing in the field, which is a prerequisite for large-scale hybrid seed production [5]. In hybrid rye, numerous CMS systems exist, of which the most predominant is the Pampa (P) type [6]. In this system, five major P-type *Rf* genes have been identified on 1RS, 4RL (*Rfp1*, *Rfp2*, *Rfp3*), and 6R (dominant modifier) chromosomes, and three minor genes on 3RL, 4RL, and 5R chromosomes [7,8,9,10]. The less prevalent CMS systems include the ‘Gülzow’ (G) type originating from the population of rye variety ‘Schlägler alt’ [11], R-type originating from a Russian population [12], and C- [13] and S- [14] types originating from the old Polish population of rye variety ‘Smolickie’. In the G-type CMS system, one major gene has been identified on 4RL (*Rfg1*) and two minor genes on 3R (*Rfg2*) and 6R (*Rfg3*) chromosomes [15]. In the C-type CMS system, two major *Rf* genes have been identified on 4RL (*Rfc1*) and 6RS (*Rfc2*) [16,17]. Intriguingly, Stojalowski et al. [18] observed a linkage between major *Rf* genes on 4RL for all three CMS systems, C-type (*Rfc1*), G-type (*Rfg1*), and P-type (*Rfp1*, *Rfp2*, *Rfp3*) to the same marker loci. This finding accentuates the pivotal importance of 4RL across CMS systems in hybrid rye breeding.

Restoration of male fertility in hybrids derived from the predominant P-type cytoplasm is frequently incomplete and highly environmentally unstable [9,19,20,21]. In addition to a potential loss in grain yield, partial pollination renders the cultivar susceptible to fungal infection by ergot (*Claviceps purpurea* (Fr.) Tul.) which can contaminate the rye grains with toxic sclerotia [22,23,24]. The P-type system is inherently shaped by the low frequency of restorer gametes in European populations in which the predominance exhibits unsatisfactory restoration [19,20]. In 1991, several non-adapted Argentinian and Iranian rye populations with a high frequency of restorer gametes were identified [25]. Crossing an elite maternal line with one of these non-adapted exotics led to observations of significantly higher restoration levels and environmental stability [26,27]. In order to steer the introgression of novel superior exotic *Rf* genes through marker-assisted selection, molecular markers were developed for *Rfp1*, *Rfp2*, and *Rfp3* [8,9]. Hybrids carrying an exotic *Rf* gene were, however, found to exhibit a significant reduction in grain yield by 4.4% to 9.4% caused by linkage drag effects or epistatic interactions associated with the exotic *Rf* gene [28]. Despite these deleterious effects, hybrid cultivars carrying the exotic *Rfp1* have been introduced to the Northern European market by a patented brand, PollenPlus^®^ [29]. In contrast, hybrids derived from the less prevalent G-type cytoplasm are characterized by a complete and environmental stable restoration of male fertility [30]. However, having received little scientific attention, the underlying genetics of the G-type CMS system remain largely unexplored [15].

The male sterility factors in CMS lines are encoded by mitochondrial genes that cause a defect in the production of viable pollen [31]. While multiple gene families have been linked with male fertility restoration, a distinct clade of the pentatricopeptide repeat (PPR) RNA-binding factor family referred to as *Rf*-like PPR (RFL-PPR) constitutes the predominant class of isolated *Rf* genes [5]. Proteins of the PPR superfamily are characterized by up to 30 tandem repeats of a canonical 35-amino-acid motif [32]. Based on motif composition, PPR genes are divided into two subclasses: the P class solely containing the canonical motif and the PLS class containing triplets of P, L (‘long’, ≈36 aa), and S (‘short’, ≈31 aa) motifs [33,34]. While the P class has predominantly been associated with RFL-PPR genes, instances of the PLS class have also been identified [35]. In rye, 591 PPR genes have been identified, out of which 83 belong to the RFL-PPR clade [36] (Table S8a). PPR proteins target mitochondrial or chloroplast mRNA, participating in a range of post-transcriptional processes (RNA editing, splicing, cleavage, and translation) with profound effects on organelle biogenesis and function [33,37,38]. In wheat, the RFL-PPR genes *Rf1* and *Rf3* have been shown to bind to a mitochondrial *orf279* transcript, induce cleavage, and prevent the expression of the sterilizing factor [39]. Within grasses, several isolated *Rf* genes have been characterized as RFL-PPRs, including *Rfm1* in barley [35], *Rf1* in sorghum (*Sorghum bicolore* L.) [40], *Rf5* in maize [41], and *Rf4*, *Rf5*, and *Rf6* in rice (*Oryza sativa* L.) [42,43,44]. In the C-type CMS system of rye, the *Rfc1* locus has been found to reside in close proximity to a cluster of RFL-PPR genes on 4RL [36].

Another gene family less prevalently associated with Rf is the mitochondrial transcription termination factors (mTERF) [45]. In rye, 131 mTERF genes have been identified [36] (Table S8b). Similar to the PPR, mTERF genes encode helical repeat proteins that target mitochondrial DNA, regulating the expression of mitochondrial genes [46]. Within grasses, mTERF genes have been associated with restoration of male fertility in barley *Rfm3* on the 6HS chromosome [47] and wheat *Rf9* on the 6BS chromosome [48]. In the P-type CMS system of rye, the *Rfp1* locus has been found to reside in close proximity to four mTERF genes [49,50]. In consistence, hotspots of RFL-PPR and mTERF genes have been identified in regions harboring known *Rf* genes in rye [36].

In this paper, we report an investigation of the genetics underlying male fertility restoration in G-type CMS-based hybrid rye breeding systems. The objective of this study was to identify major and minor G-type *Rf* genes. This was approached through: (I) genome-wide association study (GWAS) on a G-type CMS hybrid rye breeding germplasm; (II) GWAS on a biparental mapping population for studying the inheritance of male fertility restoration; and (III) gene expression analysis of PPR, RFL-PPR, and mTERF genes residing in Rf-associated blocks in two G-type hybrid cultivars for the identification of causative genes. This knowledge will serve as a stepping stone towards developing novel hybrid cultivars exhibiting superior and environmentally stable restoration of male fertility to maximize grain yield and enhance ergot resistance.

## 2. Results

### 2.1. Analysis of Genotyping Data

Prior to bioinformatic analysis using the single nucleotide polymorphism (SNP) array genotype data, a quality filtration was conducted to remove monomorphic, non-informative markers. Polymorphism information content (PIC) was calculated as a measure of the identified marker’s informativeness, with a mean PIC of 0.26 for the 20K platform (*n* = 365), 0.34 for the 30K platform (*n* = 181), and 0.23 for the 600K platform (*n* = 180). All SNP arrays portrayed a uniform distribution of markers across the rye genome (Table S1). In total, 4419 informative markers were identified in the 20K array on the entire germplasm and can be found thoroughly characterized in a recent study by Vendelbo et al. (2020). A subset of this germplasm was genotyped on the recent rye 600K array, yielding 261406 informative markers. In the F_2_ mapping population composed of 181 plants, 3493 informative markers were identified, out of which 1088 were derived from the 5K rye array, 808 from the 600K rye array, and 1597 from the 90K wheat array.

### 2.2. Genome-Wide Association Study—Case Control

**Figure 1:**
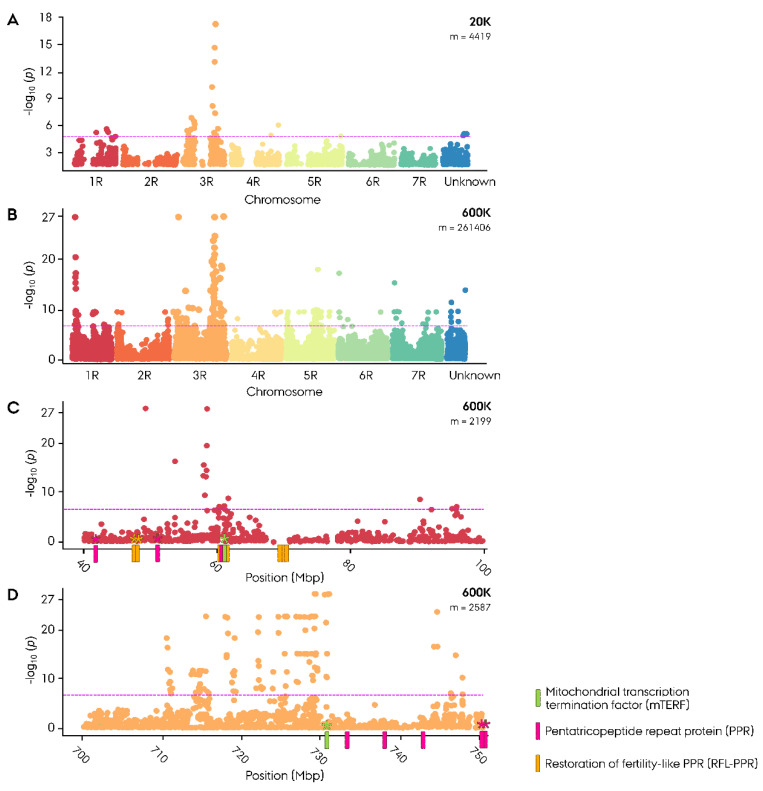
Manhattan plot for genome-wide association study (GWAS) on population origin (‘case control’) in Nordic Seed elite hybrid rye (*Secale cereale* L.) breeding germplasm. (**A**) Genome-wise Manhattan plot of 20K SNP array GWAS (*n* = 365). (**B**) Genome-wise Manhattan plot of 600K SNP array GWAS (*n* = 180). (**C**) Manhattan plot of the 600K SNP array 1RS region. (**D**) Manhattan plot of the 600K SNP array 3RL region. Position of major restoration of male fertility-associated genes has been included with genes expressed in G-type hybrids marked with an asterisk. Significant association was identified using criterion of −log_10_(*p*) > 4.95 in 20K and >6.72 in 600K depicted as a magenta line.

Genome-wide association study (GWAS) was conducted using population origin as phenotypic input in a case control analysis for an initial ‘crude’ identification of potential restoration of male fertility (Rf) genes in the germplasm. The 20K GWAS analysis produced a distinct peak in the Manhattan plot at 724 to 745 Mbp on 3RL, with the highest associated marker (−log_10_(*p*) = 19.1) located at 745 Mbp (Figure 1A, Table S2). In the successive 600K GWAS analysis, a similar peak was identified at 710–747 Mbp with the highest associated markers (−log_10_(*p*) = 27.07) located between 729 and 730 Mbp (Figure 1B–D, Table S3).

The identified Rf-associated region harbored five PLS-class PPR genes and one mTERF gene in the ‘Lo7’ reference genome (Figure 1D, Table S7a). In addition, a unique peak portraying a similarly strong association was found on 1RS at 49.3–58.5 Mbp in the 600K GWAS analysis (Figure 1C). The region harbored 22 RFL-PPR genes organized in four clusters, 2 P-class PPR genes, and 1 mTERF gene (Figure 1C, Table S7b). Out of 20 significant associated markers residing at the site, 18 mapped to a narrow peak from 58.02 to 58.47 Mbp on 1 RS (Table S3).

### 2.3. Biparental Population

**Figure 2:**
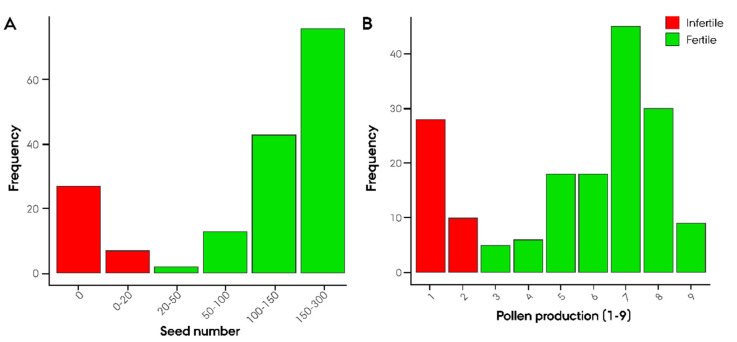
Phenotypic distribution of restoration of male-fertility-related traits. (**A**) Seed number and (**B**) pollen production in 181 F_2_ plants derived from a ‘Gülzow’ type hybrid rye (*Secale cereale* L.) cv. Stannos.

A biparental F_2_ population consisting of 181 plants was developed from the ‘Gülzow’ (G)-type hybrid cv. Stannos. The population was phenotyped for six Rf-associated traits as well as traits related to restoration in order to obtain a comprehensive dataset on the inheritance of G-type *Rf* genes. Seed number and pollen production were found, on the basis of our observations, to be the most representative Rf-associated traits (Figure 2A,B).

**Figure 3:**
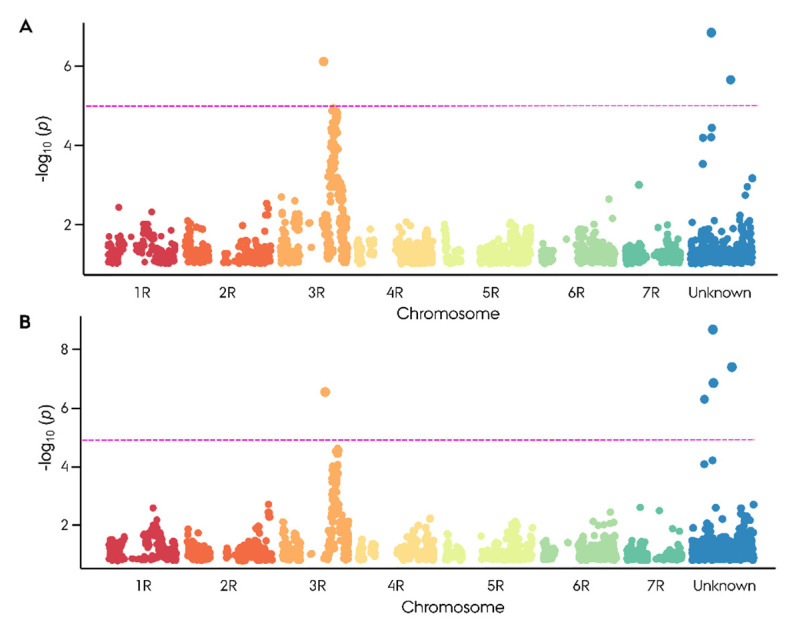
Manhattan plot for genome-wide association study on the restoration of male-fertility-related phenotypic scores. (**A**) Seed number and (**B**) pollen production (1–9) in an F_2_ biparental population composed of 181 plants derived from the hybrid rye cv. Stannos. In total, 3494 informative SNP markers were used. Significant association was identified using criterion of −log_10_(*p*) > 4.85 depicted as a magenta line.

The observed segregation ratio of sterile and fertile F_2_ plants was tested for goodness of fit to the expected Mendelian ratio at the scenario of one, two, and three major *Rf* genes using an χ^2^ test. Intriguingly, the observed segregation ratios were in accordance with a monogenic dominant inheritance of male fertility restoration with χ^2^ (1, *n*,_infertile_ = 38, *n*,_fertile_ = 143) = 2.26, *p* = 0.13 for seed number and χ^2^ (1, *n*_infertile_ = 43, *n*_fertile_ = 138) = 1.11, *p* = 0.29 for pollen production (Table S4). GWAS led to the identification of 16 Rf-associated SNP markers, of which 5 markers showed a significant association with −log_10_(*p*) > 5.2 (Figure 3). On 3RL, a twin peak was identified in the GWAS. The first peak spanning from 627 to 769 Mbp with the highest associated marker derived from the 90K wheat array (−log_10_(*p*) = 6.66) was localized at 627 Mbp. The majority of the Rf-associated markers were located around 745 Mbp (Figure 3, Table S4). The second peak, comprising four markers, spanned from 807.1 to 808.7 Mbp with its highest associated marker (−log_10_(*p*) = 4.68) just below the significance threshold of 4.85.

**Figure 4:**
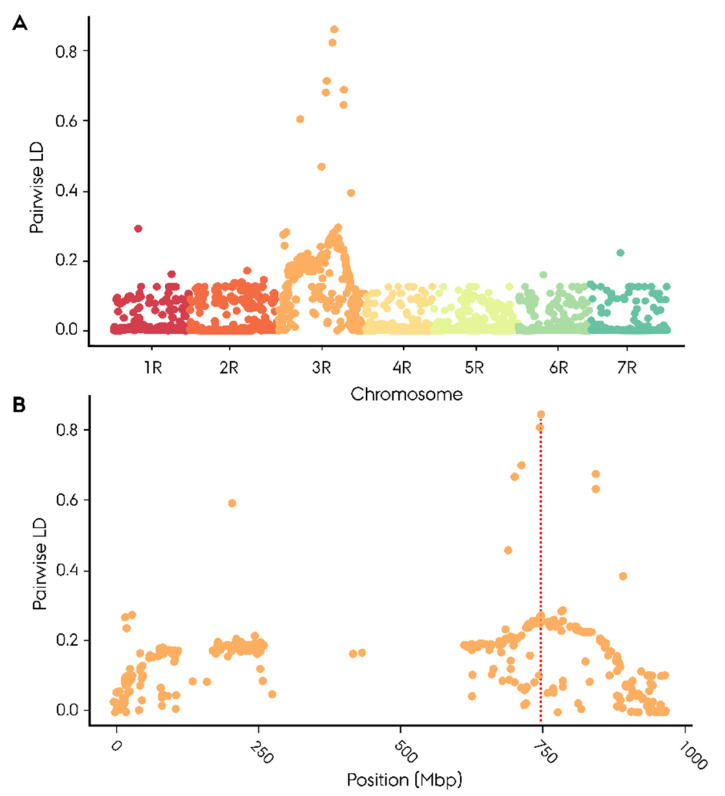
Mapping of an SNP marker derived from 90K wheat array highly associated with restoration of male fertility in 181 (*Secale cereale* L.) F_2_ plants by genome-wide pairwise linkage disequilibrium (LD) towards 2448 informative SNP markers with accurate mapping position on ‘Lo7’ rye reference genome. (**A**) Genome-wide distribution of LD, (**B**) LD distribution on 3R chromosome.

The remaining four significantly associated SNP markers were derived from the 90K wheat SNP array. None of the four markers could successfully be mapped to the ‘Lo7’ rye reference genome. Two of these markers mapped to the short arm of the wheat 3B chromosome, including the highest Rf-associated (−log_10_(*p*) = 9.12) wheat marker AX_158558079. One of the remaining moderately Rf-associated markers mapped to the short arm of the wheat 1A chromosome, while the last had no available mapping position in wheat. With no mapping position, genome-wide pairwise linkage disequilibrium was calculated for each of the four highly Rf-associated wheat-derived SNP markers to determine their position based on linkage to mapped markers (Table S5). All four wheat markers exhibited a singular peak on 3RL with a top LD ranging from 0.43 to 0.97 in the region spanning 701 to 747 Mbp (Figure S2). The top Rf-associated wheat marker AX_158558079 exhibited a max LD of 0.85 at 747 Mbp (Figure 4A,B). None of the Rf-associated wheat-derived markers showed linkage towards the Rf-associated marker cluster at 807.1–808.7 Mbp (Table S9).

### 2.4. Genomic Scan and Expression of Genes Residing within Restoration of Male-Fertility-Associated Regions of G-Type Hybrids

To identify a likely candidate *Rf* gene, a genomic scan of identified Rf-associated regions was performed and gene expression was investigated in two G-type hybrids. As the RNA-seq data were obtained from spikes at flowering, active expression of *Rf* genes would be expected in the fertile hybrids. The Rf-associated region spanning 710 to 760 Mbp was found to harbor 448 genes in the ‘Lo7’ reference genome, out of which 272 were expressed in cv. Helltop and 266 in cv. Stannis (Table S7a). Among the expressed genes, 251 were co-expressed in both hybrids. Amongst the panel of PPR, RFL-PPR and mTERF genes residing in the Rf-associated regions, and two out of five PLS-class PPR genes, at 752.1 and 759.1 Mbp, were co-expressed in both G-type hybrids (Figure 1D, Table 1). The site also harbored a single mTERF gene at 731.7 Mbp, likewise co-expressed in both G-type hybrids. The mTERF gene co-localized with the top associated marker in the 600K case control GWAS with less than 1 Mbp distance (Figure 1D, Table S3).

The Rf-associated site on 1RS spanning from 40 to 70 Mbp was found to harbor 380 genes in the ‘Lo7’ reference genome, out of which 166 were expressed in cv. Helltop and 160 in cv. (Table S7b) Stannos. Among the expressed genes, 144 were co-expressed in both G-type hybrids. Out of 13 RFL-PPR genes residing in the region, three situated at 46.2–47.1 Mbp were co-expressed in both G-type hybrids (Figure 1C, Table 1). The site also harbored two P-class PPR genes, at 42.9 and 51.9 Mbp, and a single mTERF gene, at 61.5 Mbp, likewise co-expressed in both G-type hybrids. The mTERF gene resided 3.2 Mbp from the top associated marker on 1RS.

## 3. Discussion

While the less common ‘Gülzow’ (G)-type system demonstrates superior restoration of male fertility, it has received little scientific attention in the past. This is the first study since Melz et al. [30] in 2003 to investigate the genetics underlying male fertility restoration in G-type CMS hybrid rye breeding systems. Until now, only three G-type restoration of male fertility (Rf) genes have been reported, a major gene located on 4RL (*Rfg1*) and two modifying genes on 3R (*Rfg2*) and 6R (*Rfg3*) [15,51]. By exploiting recent advents in rye genomic resources, we succeeded in identifying a novel major G-type *Rf* gene on 3RL, in addition to further evidence of a major gene on 1RS and a modifying gene on 3RL chromosome. Our findings provide a novel insight into the differentiation of the G-type fertilization control system from the predominant P-type.

### 3.1. Indications of a Major Restoration of Male-Fertility-Like Pentatricopeptide Repeat Gene on 1RS

While case control genome-wide association study (GWAS) is a useful tool for providing an insight into the genetics differentiating the parental gene pools, it has several limitations. Using population origin of lines as ‘phenotypic’ input, statistically associated markers in case control GWAS, can either be a population-defining trait such as an *Rf* QTL, or a product of population structure.

In a recent population study by Vendelbo et al. [52] on the entire G-type hybrid rye elite breeding germplasm, the maternal NRG & CMS population was found to exhibit considerable population structure and vast LD blocks. Unequal relatedness among individuals and population structure introduces a confounding effect that might cause spurious marker associations and introduce a risk of false positives [53,54]. To moderate the effect of these confounding factors, the GAPIT software used to conduct the GWAS therefore utilizes a compressed mixed linear model [55,56]. Large LD blocks, on the other hand, introduce an uncorrectable confounding factor. Long-distance LD complicates the disentanglement of actual causal variants from linked neutral markers, which can in turn lead to spurious associations [54].

In the 600K case control GWAS, a unique strong peak was identified on 1RS (Figure 1B,C). While the evidential significance of case control GWAS is insufficient to draw definitive conclusions, it provides an insight into pivotal genomic sites differentiating the parental populations. Intriguingly, the region was found to harbor 22 out of the 83 annotated *Rf*-like PPR (RFL-PPR) genes in the ‘Lo7’ reference genome situated in three large clusters (Figure 1C) [36]. In wheat, the syntenic segment has been reported to house two major *Rf* genes, *Rf1* (1AS) and *Rf3* (1BS), both belonging to the RFL-PPR gene family [39,57,58]. In rye, a major P-type *Rf* gene has likewise been identified on 1RS in a German inbred rye line ‘L18’ [7]. This *Rf* gene has, however, received little attention and it remains unknown whether the underlying *Rf* gene belongs to the RFL-PPR family.

Examining the RNA-seq data, none of the eight RFL-PPR genes residing in close proximity to the top associated marker peak were, however, found to be co-expressed at the flowering stage in the G-type hybrids (Figure 1C, Table S7b). Instead, a mitochondrial transcription termination factor (mTERF) gene residing 3.2 Mbp from the peak was found to be co-expressed.

While these findings suggest that the germplasm houses an additional major G-type *Rf* gene on 1RS, we did not observe any Rf-associated QTLs on 1RS in the mapping population GWAS (Figure 3 and Figure 4). This can either be due to the absence of the major *Rf* gene on 1RS in the pollen father of cv. Stannos, or that the region harbors a population-defining trait other than an *Rf* gene. Given that the locus aligns with a region containing numerous known Rf genes in both wheat and rye, as well as an mTERF gene co-expressed in both G-type hybrids, it is not unlikely that a large G-type Rf gene occurs on 1RS in the germplasm.

### 3.2. Modifying G-Type Restoration of Male Fertility Genes

While incapable of restoring male fertility, minor *Rf* genes are believed to enhance (‘modify’) the effect of major *Rf* genes [7]. At present, two minor *Rf* genes have been identified in the G-type CMS hybrid rye breeding systems on the 3R (*Rfg2*) and 6R (*Rfg3*) chromosomes [15]. In the mapping population, we identified an *Rf* QTL at 807.1–808.7 Mbp, indicative of a minor *Rf* gene (Table S4). While no positional information exists on the *Rfg2* gene, a minor P-type *Rf* gene has been identified in the German inbred line ‘L18’ with associated markers mapping to 806.1 and 869.5 Mbp [7]. Calculation of pair-wise LD ruled out the presence of spurious association between the potential minor *Rf* gene and the downstream major *Rf* gene, confirming an independent QTL (Table S9). It remains uncertain whether the potential minor *Rf* gene represents a unique gene or the previously described *Rfg2*.

### 3.3. Decisive Role of 3R in the G-Type CMS Breeding System

The initial genome scan for population-differentiating traits using case control GWAS indicated that the 3R chromosome played a unique role in the G-type CMS system (Figure 1A,B). This is consistent with previous population studies on the assayed germplasm, identifying a singular enrichment of interchromosomal LD for both parental populations on the 3R chromosome, suggesting a conservation of a population-defining trait or traits [52].

To investigate whether the population-differentiating region on 3RL harbored a G-type *Rf* gene, a biparental mapping population was developed. In contrast to the case control GWAS, the biparental mapping population is not subject to confounding issues related to population structure. The segregation ratio of Rf-associated traits in the mapping population was found to be in accordance with a monogenic dominant inheritance of an *Rf* gene by χ^2^ test consistent with the singular peak identified in the case control GWAS (Figure 1A,B). Since minor *Rf* genes are incapable of restoring male fertility on their own, they do not influence the segregation ratio of infertile/fertile F_2_ progeny and are hence not ‘caught’ in the χ^2^ test. Utilizing the phenotypic dataset on Rf-associated traits from the mapping population, we identified an Rf-associated region on 3RL consistent with findings in the initial case control GWAS (Figure 3A,B). The precise position of the causative *Rf* gene was, however, initially obscured by the finding that the four most associated SNP markers, deriving from the 90K wheat array, could not be accurately mapped to the rye reference genome ‘Lo7’ [36,59]. Instead, chromosome-wide LD mapping of each Rf-associated wheat marker was conducted, with the top associated marker mapping to 747 Mbp (Figure 4, Table S5). Intriguingly, while the 4R chromosome plays a pivotal role in the P- and C-type CMS systems in rye, housing *Rfp1*, *Rfp2*, *Rfp3*, and *Rfc1*, we found no evidence of G-type genes on 4R in either case control or mapping population GWAS (Figure 1 and Figure 3) [8,9,28,60]. Instead, our findings suggest that the 3R chromosome plays a unique role in the G-type CMS system, possibly harboring both a minor and major *Rf* gene.

### 3.4. Novel Major Restoration of Male Fertility Gene Unique to the G-Type CMS Breeding System

While no major *Rf* gene on the 3RL chromosome has to our knowledge been identified in either of the known CMS systems in rye, minor genes have been identified in both the G-type and P-type [7,15]. In the case of *Rfg2*, ambiguous segregation ratios of primary trisomics of rye 3R led to the assumption that 3R likely housed a minor G-type *Rf* gene. While Melz and Adolf [15] cautiously interpreted this anomaly as a product of a modifying gene, their findings suggest that something of significance is occurring on 3R in the G-type CMS system. It therefore remains open whether the identified major G-type gene is *Rfg2*, previously misclassified as a minor gene, or an unreported *Rf* gene on 3RL. We propose to denote the major G-type *Rf* gene on 3RL *RfNOS1.*

To our knowledge, no *Rf* gene has been reported on chromosome segments orthologous to rye 3RL in any of the domesticated species residing within the botanical tribe Triticeae. In wheat, major *Rf* genes have been identified on 1AS (*Rf1*), 1BS (*Rf3*), 6AS (*Rf9*), 6BS (*Rf4*, *Rf6*), 6D (*Rf5*), and 7D (*Rf2*) chromosomes [48,57,58,61,62,63]. In barley (*Hordeum vulgare* L.), two major *Rf* genes have been identified on 6HS (*Rfm1*, *Rfm3*) [47,64]. Intriguingly, Martis et al. [65] discovered that the distal regions of 3RL and 4RL conserved syntenic segments of an ancestral Triticeae chromosome a6. In a comparative analysis, they found that the segment on 3RL portrayed distinctly less collinearity than all other syntenic segments, suggesting a differential evolution of 3RL during rye speciation. On the contrary, the syntenic segment on 4RL was found to be highly conserved in *Brachypodium distachyon* L., rice (*Oryza sativa* L.), sorghum (*Sorghum bicolore* L.), and barley. Rye 4RL, a region housing three major P-type and one C-type *Rf* genes, was found to be syntenic to barley 6HS [60,65]. These results are consistent with the findings of Hackauf et al. [50], who reported that the segment housing *Rfp1* on 4RL exhibits an ortholog on wheat 6DS and barley 6H. In a subsequent study, Hackauf et al. [8], furthermore, proposed that *Rfp3* on 4RL likely maps to an orthologous segment housing *Rf6* on wheat 6BS and *Rfm1* on barley 6HS [62]. These findings provide further evidence of a conserved synteny between these chromosomal segments and the conservation of *Rf* genes pivotal to the fertilization control systems across rye (P- and C-type), barley, and wheat [51]. Together, this accentuates the novelty of our discovery of a major *Rf* gene, *RfNOS1*, on 3RL in the G-type CMS system in rye, with no known *Rf* genes on orthologous chromosome segments in other Triticeae species.

### 3.5. Non-Pentatricopeptide Repeat Restoration of Male Fertility Gene on 3RL

The majority of characterized *Rf* genes have been assigned to the PPR superfamily, denoted as *Rf*-like PPR genes or RFL-PPRs. In domesticated Poaceae species, this includes wheat *Rf1* and *Rf3* [39], barley *Rfm1* [35], sorghum *Rf1* [40], maize *Rf5* [41], and rice *Rf4*, *Rf5*, and *Rf6* [42,43,44]. A genome scan for Rf-associated genes at the site on 3RL harboring the major G-type *Rf* gene *RfNOS1* in the ‘Lo7’ reference genome revealed no RFL-PPR genes. Instead, the region harbored five PLS-class PPR genes either misclassified, not resembling known PLS-type RFL-PPR genes, or pointing towards a non-PPR *Rf* gene on 3RL (Figure 1D, Table S8a). Out of 83 RFL-PPRs annotated in the ‘Lo7’ reference genome, none of them belong to the PLS class. Whereas a less prevalent group, PLS-type RFL-PPR genes exists, as observed in the case of *Rfm1* in barley [35]. It, therefore, cannot be ruled out that the RFL-PPR annotation of ‘Lo7’ is incomplete, inclined to annotate the prevalent P-class as RFL-PPR genes, while disregarding the less well-represented PLS class. However, both of the PLS-class PPR genes residing in the Rf-associated region on 3RL that were co-expressed in the two G-type hybrids resided more than 20 Mbp from the top associated peak in the 600K case control GWAS (Figure 1D, Table S8b). In conjunction, this suggests that the major *Rf* gene on 3RL belongs to the non-PPR family.

A growing body of *Rf* genes is now being characterized as non-PPR *Rf* genes, adding to the complexity of male fertility restoration. Until now, this includes glycine-rich proteins (Rf2, in rice; [66]), acyl-carrier protein synthase (Rf17, in rice; [67]), aldehyde dehydrogenase (Rf2, in maize; [68]), bHLH transcription factor (Rf4, in maize; [69]), and *Rf1*, a peptidase, in sugar beet (*Beta vulgaris* L.) [70]. The region on 3RL harbored several genes related to plant fertility that were co-expressed in both G-type hybrids at anthesis (Table S7a). Amongst these were two KATANIN genes located at 730.3 and 759.9 Mbp. In *Arabidopsis*, KATANIN is required for fertility, embryo development, and seed production [71]. Genes involved in flower development, including MADS-box transcription factor [72,73,74], BZIP transcription factor [75], and basic helix-loop-helix (bHLH) DNA-binding superfamily protein [69] were likewise found co-expressed within the region.

Intriguingly, the region was found to harbor a single mTERF gene located at 731 Mbp, coinciding with the top-associated peak in the 600K case control GWAS (Figure 1D, Table S8b). In rye, Hackauf et al. [8] reported a close linkage between *Rfp1* and *Rfp3* on 4RL to mTERF genes. A similar observation was made by Bernhard et al. [47], who identified two mTERF genes closely linked to *Rfm3* on barley 6HS, syntenic to rye 4RL. Pan et al. [76] successively observed the role of mTERF genes in kernel development in maize, connecting the gene family to the reproductive system of plants. This newly identified mTERF gene, which is expressed at flowering in both G-type hybrids, is a potential candidate for the novel major G-type *Rf* gene on 3RL. We denote the gene as *RfNOS1*, belonging to the expanding class of non-PPR *Rf* genes.

### 3.6. CMS Systems in Hybrid Rye Breeding

On the basis of male fertility restoration requirements and genetic similarity of sterilizing cytoplasms, G-, C-, and R-type CMS systems have been proposed to belong to the larger Vavilovii (V) type [6,77,78]. In a comprehensive study by Lapinski and Stojalowski [6] on 50 rye populations from 23 countries, the vast majority of male sterility sources were found to belong to the V-type. Populations with European descent were predominantly found to carry the V-type sterility-inducing cytoplasm, while the P-type was exclusively observed in lines descending from South America. Nonetheless, with no previous report of a major *Rf* gene on 3RL in either R, S, or C-type CMS system, such a unilateral grouping as V-type seems premature. From our observations, the G-type CMS system distinguishes itself from the other CMS systems by a less pivotal role of *Rf* genes on 4RL.

## 4. Methods

### 4.1. Plant Material

In total, 365 Nordic Seed Germany GmbH inbred hybrid rye (*Secale cereale* L.) elite breeding lines were selected for this study, comprising 242 restorers, 116 non-restorer germplasm (NRG), and 7 cytoplasmic male sterile (CMS) lines. The CMS male sterility is based on the ‘Gülzow’ (G)-type cytoplasm originating from the population of the rye variety ‘Schlägler alt’ [11,15]. Population structure and information on genetic characteristics of the germplasm are presented in a recent study by Vendelbo et al. [52]. A biparental mapping population was developed from a hybrid rye cv. Stannos. DNA extraction was performed using an adapted SDS-based method according to USDA [79] after Pallotta et al. [80] on an equivalent of 75 mg of plant material collected from the primary leaves of two seven-day-old, seedlings per line. DNA concentration and 260/280 nm ratio of samples were measured using an Epoch^TM^ microplate spectrophotometer (Biotek^®^ Vermont, Winooski, VT, USA) and evidence of fragmentation by size visualization on a 1.2% agarose gel.

### 4.2. Biparental Mapping Population

To investigate the inheritance of male fertility restoration in the G-type CMS-based Nordic Seed breeding system, a biparental mapping population was developed. The population was phenotyped for restoration of male fertility, including traits associated with restoration. Seeds of the hybrid cv. Stannos (F_1_) were sown in pots containing a course-grain sphagnum substrate at Nordic Seed Germany GmbH greenhouse facilities. The seedlings were cultivated under a 16 h light regime with night temperatures of 14–16 °C and day temperatures of 18–24 °C. Seven days after sowing, at the 2-leaf stage, seedlings were set to vernalize in a climate chamber under 16 h of light at 8 °C for a week and then 3 °C for the following seven weeks. After vernalization, the pots were transferred to the greenhouse. Prior to anther-protrusion, cellophane bags were put on the spikes to prevent cross-fertilization. At maturity, seeds of a single F_1_ plant were harvested and the procedure was repeated to generate an F_2_ biparental mapping population. At four timepoints, a total of 181 F2 plants were phenotyped for pollen production using a customized visual 1–9 scale (1: no pollen, 9: large amount of pollen). The plants were, furthermore, scored for number of spikes per plant, total seed number, seeds per spike, total grain weight, and thousand kernel weight in order to obtain a comprehensive phenotypic dataset on the inheritance of male fertility restoration in the population. The segregation ratio of infertile and fertile F_2_ plants was tested for goodness of fit to the expected Mendelian ratio at the scenario of one, two, and three major restorations of male fertility (*Rf*) genes using an χ^2^ test [81]. An F_2_ plant was considered ‘sterile’ if it either yielded less than 20 seeds or scored ≤ 2 in pollen production.

### 4.3. Molecular Markers

All rye lines included in this investigation were genotyped using a custom Illumina Infinium 15K_wheat_ [59] and 5K_Rye_ [82,83] single nucleotide polymorphism (SNP) array, denoted 20K, as described by Vendelbo et al. (2020). In addition, 180 lines comprising 88 NRG and 92 restorer lines were also genotyped using the state-of-the-art 600K high-density rye array by Bauer et al. [83]. The F_2_ biparental mapping population was genotyped on a custom Illumina Infinium 25K_wheat_ and 5K_Rye_ SNP array, denoted 30K, enriched with an additional 10K wheat markers, compared to the previous 20K array, deriving from the 90K wheat SNP array by Wang et al. [59]. The mapping position of SNP markers derived from the 90K wheat array was found by mapping the marker sequences to the ‘Lo7’ reference genome using the NCBI blastn (v. 2.9.0+, ML, USA) function at a significance threshold of the e-value at 10^−5^, selecting the physical position of the top hit [36,84]. The position of *Rf*-associated markers without accurate mapping position in the ‘Lo7’ reference genome was determined by calculation of pairwise LD across the entire mapped marker panel. LD was calculated using snpStats (v. 1.36.0, Cambridge, UK) R package with LD set as the coefficient of determination (r^2^) [85].

### 4.4. Data Analysis

Genetic analysis of SNP marker data was performed in R studio (v. 1.1.463, Boston, MA, USA) interface in R statistical software (v. 3.6.3) by application of various predesigned packages [86,87]. Prior to analysis, markers were filtered for marker allele frequency ≥0.005, missing individual score ≤ 0.2 and missing marker score ≤ 0.1 to identify informative markers. The polymorphism information content (PIC) was calculated as an estimate of marker informativeness using SnpReady (v. 0.9.6, Laguna, Philippines) [88].

### 4.5. Genome-Wide Association Study

The discovery of *Rf*-associated regions was made by a genome-wide association study (GWAS) using the genomic association and prediction integration tool (GAPIT) (v.3) package in R [55]. Phenotypic input for GWAS included all recordings of the biparental F_2_ population, and a binary case control for the entire population relative to their population origin using the 20K SNP array and 600K high-density SNP array, respectively. A standard Bonferroni-corrected threshold of α = 0.05 was used as the significance threshold

### 4.6. RNA-Seq Data Expression Analysis of PPR and mTERF Genes Residing in Rf-Associated Region in G-Type Hybrids of Rye

Annotated major *Rf*-associated genes, pentatricopeptide repeat proteins (PPR), Rf-like PPR, and mitochondrial transcription termination factors (mTERF), residing in regions associated with Rf were identified in the ‘Lo7’ reference genome [36]. To ascertain the potential causative Rf gene or genes amongst the identified panel, gene expression was investigated in two G-type Nordic Seed hybrid cv. Helltop and cv. Stannos de novo transcriptome assemblies. The transcript data were obtained from spikes of the G-type hybrids at flowering. High-quality de novo transcriptome assembly of the two hybrids has recently been published by Mahmood et al. [89] and raw reads from the transcript library have been made accessible in a sequence read archive repository at (https://www.ncbi.nlm.nih.gov/bioproject/PRJNA612415, accessed on 12 March 2021). Coding and protein sequences of the Rf-associated gene panel were extracted from the ‘Lo7’ reference genome repository. The de novo assembled transcriptome assemblies were likewise translated to coding protein sequences with TransDecoder (v. 5.5.0, Valencia, Spain). The NCBI blastx and blastp (v. 2.9.0+, Maryland, USA) functions were used to blast coding and protein sequences of Rf-associated genes in the panel towards the generated protein transcriptome database at a significance threshold of the e-value at 10^−5^ to ascertain genes expressed in the two G-type hybrid cv. at flowering stage [84].

### 4.7. Graphical Editing

Graphs and figures were outputted from R in .svg format and manually curated using Inkscape (v. 1.1) program (https://inkscape.org/, accessed on 7 May 2021).

## 5. Conclusions

In this study, we exploited recent advents in rye genomic resources to dissect the genetics underlying restoration of male fertility in a G-type CMS system. Our findings provide compelling evidence of a novel major G-type *Rf* gene on 3RL with no known orthologues in either barley or wheat. Gene mining of the Rf-associated region on 3RL led to the identification of an mTERF gene co-expressed in two G-type hybrids as a candidate gene for restoration. We propose to denote the novel G-type *Rf* gene as *RfNOS1*. Conclusively, our investigation provides a novel insight into the genetics of male fertility restoration in a G-type CMS system and its differentiation to rye P- and C-types in addition to known CMS systems in barley and wheat.

## Figures and Tables

**Table 1:** Pentatricopeptide repeat protein (PPR) and mitochondrial transcription termination factor (mTERF) genes in restoration of male-fertility-associated regions identified on 1RS (40–70 Mbp) and 3RL (710–760 Mbp) chromosome in ‘Lo7’ rye (*Secale cereale* L.) reference genome. Genes expressed in ‘Gülzow’ type hybrid cultivars at flowering are marked as green, and genes not expressed as red.

Chromosome	Position (Mbp)	Gene Length (bp)	Annotation	Expression	
				cv. Helltop	cv. Stannos
1R	42.9	822	PPR (P Type)		
	46.2	2091	RFL-PPR (P Type)		
	46.2	2613	RFL-PPR (P Type)		
	46.3	2601	RFL-PPR (P Type)		
	46.9	2487	RFL-PPR (P Type)		
	47.1	2490	RFL-PPR (P Type)		
	47.1	1833	RFL-PPR (P Type)		
	51.9	1659	PPR (P Type)		
	61.1	417	RFL-PPR (P Type)		
	61.3	287	PPR (P Type)		
	61.3	213	PPR (P Type)		
	61.5	2499	RFL-PPR (P Type)		
	61.5	1866	mTERF		
	61.8	2472	RFL-PPR (P Type)		
	61.9	2448	RFL-PPR (P Type)		
	61.9	1460	RFL-PPR (P Type)		
	61.9	2451	RFL-PPR (P Type)		
	62.0	2499	RFL-PPR (P Type)		
	70.8	510	mTERF-like		
	70.8	540	mTERF-like		
3R	731.7	462	mTERF		
	733.4	1986	PPR (PLS Type)		
	738.4	1743	PPR (PLS Type)		
	743.7	1461	PPR (PLS Type)		
	751.6	1356	PPR (PLS Type)		
	752.1	1548	PPR (PLS Type)		
	759.7	825	PPR (PLS Type)		

## Data Availability

The data presented in this study are available in the Supplementary Material.

## Supplementary Materials

The following are available online at https://www.mdpi.com/article/10.3390/ijms22179277/s1.
