# Supplementary figures and images for: Genomic Scan of Male Fertility Restoration Genes in a ‘Gülzow’ Type Hybrid Breeding System of Rye (Secale cereale L.)
Source: Int J Mol Sci. 2021 Aug 27;22(17):9277. doi: 10.3390/ijms22179277 (PMC8431178; doi:10.3390/ijms22179277)

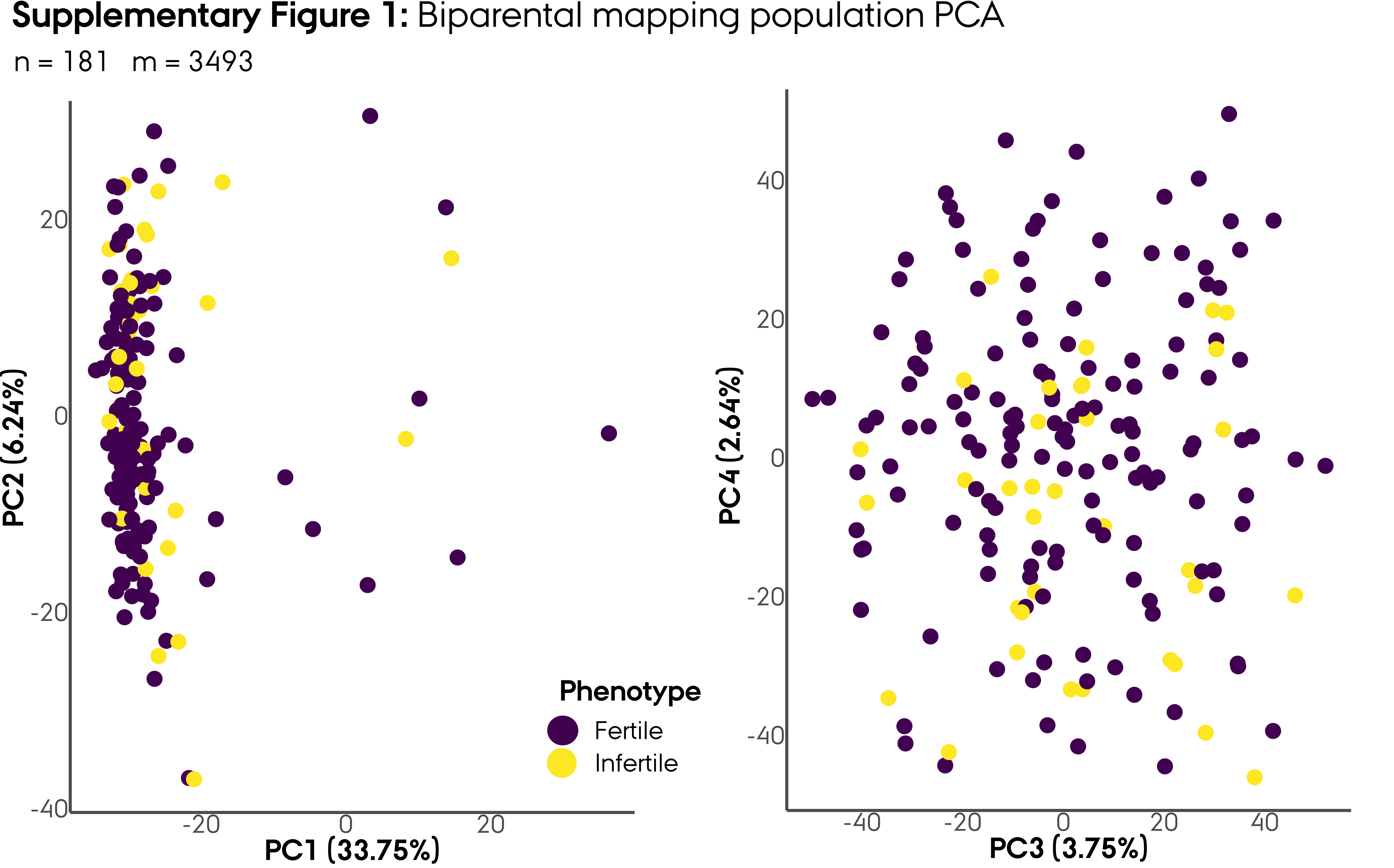

Supplement: Supplementary file 1 [file ijms-22-09277-s001.zip › Supplementary material/Figure S1.png]
